# Full-Length Transcriptomics Reveals Complex Molecular Mechanism of Salt Tolerance in *Bromus inermis* L.

**DOI:** 10.3389/fpls.2022.917338

**Published:** 2022-06-09

**Authors:** Qian Li, Jiaxing Song, Yi Zhou, Yingxia Chen, Lei Zhang, Yongzhen Pang, Bo Zhang

**Affiliations:** ^1^Key Laboratory of Grassland Resources and Ecology of Western Arid Region, Ministry of Education, College of Grassland Science, Xinjiang Agricultural University, Urumqi, China; ^2^Key Laboratory of Grassland Resources and Ecology of Xinjiang, College of Grassland Science, Xinjiang Agricultural University, Urumqi, China; ^3^Institute of Animal Science, Chinese Academy of Agricultural Sciences, Beijing, China; ^4^College of Grassland Agriculture, Northwest A&F University, Yangling, China; ^5^School of Agriculture Food and Wine, The University of Adelaide, Urrbrae, SA, Australia

**Keywords:** smooth bromegrass, full-length transcriptomics, salt tolerance, ion signal transduction, SOS regulatory pathways

## Abstract

*Bromus inermis* L. (commonly known as smooth bromegrass) is a grass species with high nutritional value, great palatability, cold tolerance, and grazing resistance, which has been widely cultivated for pasture and sand fixation in northern and northwestern China. Salt stress is a main environmental factor limiting growth and production of smooth bromegrass. In this study, we performed PacBio Iso-Seq to construct the first full-length transcriptome database for smooth bromegrass under 300 mM NaCl treatment at different time points. Third-generation full-length transcriptome sequencing yielded 19.67 G polymerase read bases, which were assembled into 355,836 full-length transcripts with an average length of 2,542 bp. A total of 116,578 differentially expressed genes were obtained by comparing the results of third-generation sequencing and second-generation sequencing. GO and KEGG enrichment analyses revealed that multiple pathways were differently activated in leaves and roots. In particular, a number of genes participating in the molecular network of plant signal perception, signal transduction, transcription regulation, antioxidant defense, and ion regulation were affected by NaCl treatment. In particular, the CBL-CIPK, MAPK, ABA signaling network, and SOS core regulatory pathways of Ca^2+^ signal transduction were activated to regulate salt stress response. In addition, the expression patterns of 10 salt-responsive genes were validated by quantitative real-time PCR, which were consistent with those detected by RNA-Seq. Our results reveal the molecular regulation of smooth bromegrass in response to salt stress, which are important for further investigation of critical salt responsive genes and molecular breeding of salt-tolerant smooth bromegrass.

## Introduction

The salt content of the soil surface is high in arid and semi-arid areas due to low precipitation, high evaporation, and inadequate water resource management (Rengasamy, 2010). Soil salinity exists in almost every irrigated area in the world, as well as in non-irrigated farmland and pasture (Ivushkin et al., 2019; Kearl et al., 2019). High content of salt in the soil causes dehydration of plant roots, imbalance of intracellular osmotic regulation, and osmotic stress. In addition, salt stress affects biological functions in plant cells, for example, inhibiting its ability to uptake water and nutrients, accelerating chlorophyll degradation, inhibiting photosynthesis, and blocking various metabolic reactions (Karan and Subudhi, 2012; Deinlein et al., 2014; Al-Farsi et al., 2020). The most direct damage is high osmotic pressure resulting from low water use efficiency and ionic toxicity (mainly Na^+^) (Chinnusamy et al., 2006). Plants generally respond to salt stress through various mechanisms, including osmotic adjustment, ion transmembrane transport, ion compartmentalization, and active oxygen scavenging (ROS) (Liu et al., 2021).

In response to high salt stress, plants have evolved a number of signal transduction pathways involving regulatory genes and proteins for stress perception, stress signaling, and downstream metabolites (Bhattarai et al., 2020). Under salt stress, various stress signal receptors located on the cell membrane can quickly perceive changes in the external environment, and second messengers such as Ca^2+^, ROS, and phytohormone are rapidly generated in the cytoplasm to gradually decode and amplify salt stress signals (Fahad et al., 2014; Steinhorst and Kudla, 2019). These signals regulate downstream TFs through a cascade reaction to further alter transcript levels of many TF genes, such as *AP2/ERF, bZIP, bHLH, WRKY, MYB, NAC*, and *DREB* (Zhao et al., 2020). Thereafter, the expression of various osmotic stress-responsive genes (e.g., *P5CSs* and *P5CRs*) and ionic stress-responsive genes (e.g., *HKTs, AKTs*, and *NSCCs*) is eventually affected and ultimately contributes to plant salt tolerance (Zhu, 2016). Several signaling pathways involved in salt stress response have been revealed, including calcineurin B-like protein (CBL)–interacting protein kinase (CBL-CIPK), mitogen-activated protein kinase (MAPK), SOS, plant hormone (e.g., ABA, Eth, BR, and GAs), and calcium-dependent protein kinase (CDPK) pathways (Zhang et al., 2020; Zhao et al., 2020).

Smooth bromegrass is a perennial fine pasture of the grass family, which is widely used as hay, silage, and pasture for ruminants and dairy production (Ferdinandez and Coulman, 2001; Smart et al., 2006). It is mainly cultivated in northern China. However, due to climatic factors and poor agricultural management, soil salinization in these regions is becoming worse, which limits the production and utilization of smooth bromegrass (Wang and Yan, 2013 Wang et al., 2021). Therefore, it is important to explore molecular mechanisms of smooth bromegrass under salt stress and identify valuable resources for further breeding programs.

Currently, next-generation sequencing (NGS) provides an accurate and comprehensive analysis of differentially expressed genes and has made significant progress in understanding plant responses to drought and salt stress. NGS based on Illumina sequencing technology has been applied in *Spartina pectinata* (Robben and Gonzalez, 2016), *Hordeum vulgare* (Zhang et al., 2021), *Spartina alterniflora* (Hana, 2015), and *Sorghum bicolor* (Cui et al., 2018) to explore the molecular response mechanism under salt stress. Meanwhile, the single-molecule real-time (SMRT) sequencing provides third-generation sequencing (TGS) technology, which outperforms NGS technology in terms of read length (Rhoads and Au, 2015). In comparison to standard NGS technology, PacBio RSII TGS employs SMRT isoform sequencing (Iso-Seq), which provides longer read length, uniform coverage, and high accuracy (Dong et al., 2015). Therefore, SMRT sequencing technology has been used in *Zea mays* (Wang et al., 2016a), *Sorghum bicolor* (Abdel-Ghany et al., 2016), *Medicago sativa* (Luo et al., 2019), and *Arabidopsis pumila* (Yang et al., 2018) to explore how plants respond to stress.

In this study, we combined NGS and SMRT sequencing to generate a full-length transcriptome in smooth bromegrass. We analyzed the expression profile of differentially expressed genes in leaves and roots of smooth bromegrass under salt stress. In addition, we also identified a set of key genes that are likely involved in salinity adaptation in smooth bromegrass. These sequencing data will facilitate future research on gene function characterization and salinity adaptation mechanism in smooth bromegrass.

## Materials and Methods

### Plant Material and Growth

*Bromus inermis* cv. Wusu No. 1 is a variety that is widely cultivated at Qitai Grassland Station in Xinjiang. It possesses characteristics of high yield, superior drought tolerance, cold resistance, rapid regeneration, early greening, and disease resistance. The full-grained seeds of Wusu No. 1 smooth bromegrass were sterilized and placed into a 26°C/16°C (light/dark, 8h/16h) light incubator for germination. At the two-leaf stage, the seedlings were transplanted into soil containing vermiculite and perlite at a ratio of 3:1 and grew in a greenhouse. The plants were irrigated with a fresh 1/2 Hoagland solution every 3 days. When the seedlings grew to the 4-leaf stage, they were selected and carefully transplanted into a triangular flask containing a 1/2 Hoagland solution to recover for 3 days. These seedlings were then treated with a 1/2 Hoagland solution containing 300 mM NaCl, and leaves and roots from 10 seedlings (with a triplicate) were sampled at 0 h, 12 h, 24 h, 36 h, and 48 h, frozen in liquid nitrogen, and stored at −80 °C.

### Analysis on Physiological Index

Chlorophyll was measured with a SPAD-502 chlorophyll meter, and the SPAD value was recorded with 10 repeats. A handy PEA portable plant fluorometer was used to determine the maximum photochemical efficiency. A DDS-11A conductivity meter was utilized to measure relative conductivity, and the relative water content method was used to measure fresh weight (FW) immediately after harvest. The plants were placed in deionized water at 4°C for 8 h. The weight (TW) saturated with water absorption was measured, dried at 105°C for 10 min, and moved to 80°C for 24 h. The dry weight (DW) of the plants was measured, and the RWC was calculated using the following formula: RWC (%) = (FW-DW)/(TW-DW) × 100. The parameters of MDA, proline, SOD, and GSH content were determined by using kits developed by Nanjing Jiancheng Bioengineering Research Institute Co., Ltd. Measurement methods were referred to the instructions of the plant malondialdehyde (MDA) assay kit (A003-3-1), proline assay kit (A107-1-1), superoxide dismutase (SOD) assay kit (A001-3-2), and reduced glutathione (GSH) assay kit (A006-2-1), respectively, as previously reported (Wang et al., 2016b, 2019; Meng et al., 2020).

### Sequencing, Assembly, and Annotation of SMRT Library

RNAs were extracted by using an EasyPure Plant RNA Kit (No. ER301), and a Nanodrop 2000 spectrophotometer was used to detect the purity of RNA. A SMARTer PCR cDNA Synthesis Kit was employed for reverse transcription. The PacBio Sequel platform (Pacific Biosciences: http://www.pacb.com) was used for sequencing, and the PacBio official software package SMRTlink was used to process the original offline data. The subread sequence and the circular consensus sequence (CCS) were produced by correcting the subreads, and the sequences were then classified as full-length or non-full-length based on the presence of 5'-end primer, 3'-end primer, or poly-A tail. The full-length sequences were then clustered using ICE to get the cluster consensus sequence. In order to measure the correlation and similarity among samples, the PCA and Pearson correlation coefficient (R^2^) were analyzed by using the R package.

We used LoRDEC software (Leena and Eric, 2014) to analyze and correct the second-generation data and third-generation PacBio data. The corrected transcripts were sequenced and clustered by CD-HIT software (Fu et al., 2012), and redundant and similar sequences were removed. CDS predictive analysis was performed using ANGEL software (Shimizu et al., 2006), and the fault-tolerant mode was adopted by default. We compared all predicted protein coding sequences with protein and nucleotide databases using BLASTX with the following databases: NR, Nt, Pfam, KOG/COG, SWISS-PROT, KEGG, and GO. The cutoff E-value for NCBI non-redundant protein (NR) and NCBI non-redundant nucleotide (NT) was ≤ 10^−5^, and that for the protein family was E-value ≤ 10^−5^. The cutoff E-values were all ≤ 10^−5^ for KOG (http://www.ncbi.nlm.nih.gov/COG/), SWISS-PROT (Amos and Rolf, 2000), and KEGG analyses (http://www.genome.jp/kegg/) (Minoru et al., 2004). The setting parameters for Gene Ontology were referred to Ashburner et al. (http://www.geneontology.org/) (Ashburner et al., 2000), and GO enrichment analysis was performed by using agriGO v2.0 (*p*-value ≤ 0.05) (Tian et al., 2017). All differentially expressed genes were analyzed by using iTAK for online plant transcription factor prediction (http://itak.feilab.net/cgi-bin/itak/index.cgi) (Yi et al., 2016).

### Illumina Sequencing and Sequence Assembly

Magnetic beads with oligo (dT) were used to enrich mRNA, and six-base random primers (random hexamers) were employed to synthesize cDNA. The double-stranded cDNAs were purified with AMPure XP beads, and PCR amplification was performed to construct the cDNA library. An Agilent 2,100 Bioanalyzer was used to detect the insert size of the library. After qualification, the Illumina second-generation high-throughput sequencing platform was used, and raw reads were filtered to obtain clean reads. Trinity (Grabherr et al., 2011) was used to assemble the transcripts of clean reads, and software RSEM (Dewey and Li, 2011) was used to calculate the gene expression using the FPKM method (Trapnell et al., 2010). Based on the average FPKM values under each treatment in roots and leaves, both the absolute values of the |log_2_ (fold change)| ≥1 and the adjusted *P*-value < 0.05 were used as thresholds to identify DEGs.

### RT-qPCR Analysis

In order to verify the accuracy of the sequencing results, 10 differential genes were selected for RT-qPCR analysis. The TransScript One-Step gDNA Removal and cDNA Synthesis Super-Mix Reverse Transcription Kit were used for reverse transcription. Total RNAs extracted from leaves and roots from the control and salt treatment groups were used to synthesize cDNA. Primer Premier 5 software was used to design primers (Supplementary Table S1) (Regina et al., 2010; Bahrini et al., 2011). Real-time PCR was performed using the Applied Biosystems 7500/7500 fast real-time PCR system (ABI, Foster City, California, USA) and the SYBR Green PCR Master Mix system (Takara). We referred to the Trans-Start Top Green qPCR Super-Mix manual for RT-qPCR with triplicates. The 2^−ΔΔCt^ method (Livak and Schmittgen, 2001) was used to quantitatively calculate the relative expression level of candidate genes.

### Statistical Analysis

Statistical data analysis was performed using Excel 2016 software and the SPSS software package (ver. 22.0; SPSS Inc., Chicago, IL, USA). MEV 4.9 software (https://sourceforge.net/pro-jects/mev-tm4/files/mev-tm4/) was used for cluster analysis and expression pattern analysis.

## Results

### Effects of Salt Treatment on Physiological Changes of Smooth Bromegrass

**Figure 1:**
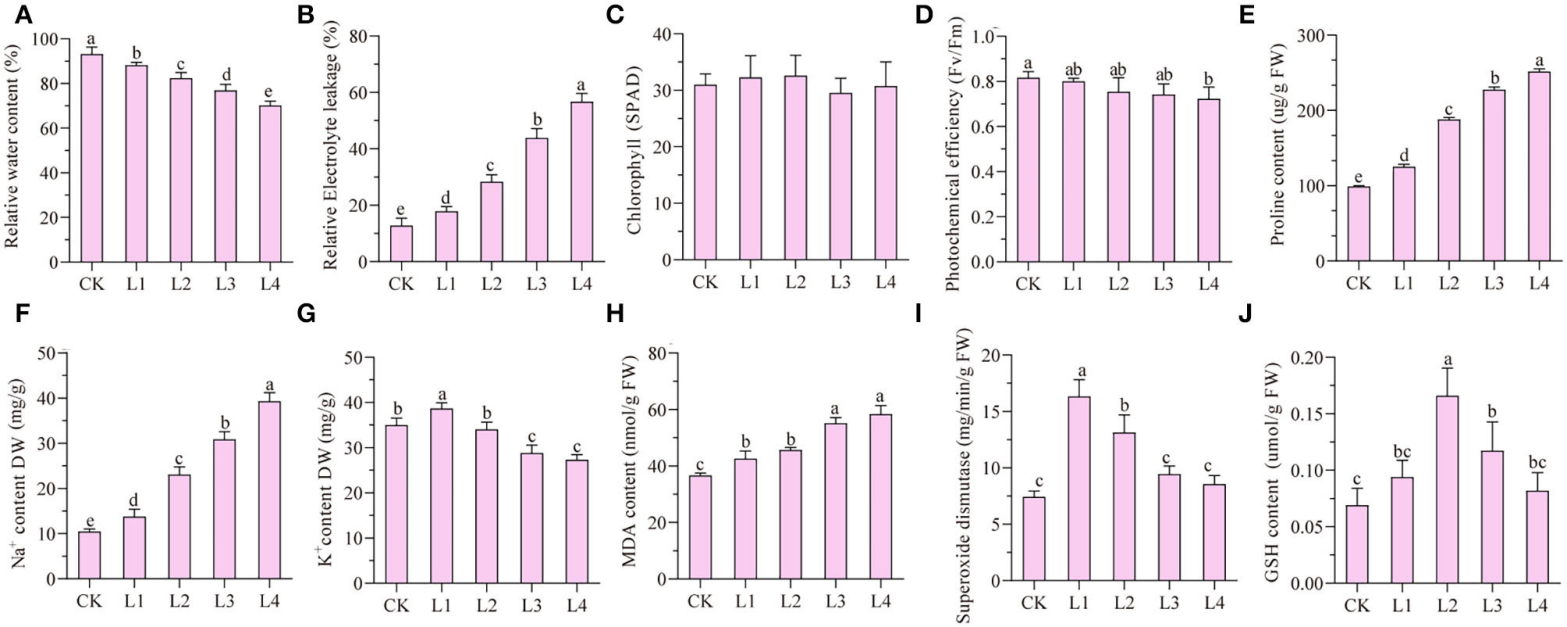
Analyses of dynamic physiological effects under 300 mM NaCl stress in the leaves. CK: untreated control; L1: salt stress treatment for 12h; L2: 24 h; L3: 36 h; L4: 48 h. **(A)** rRelative water content; **(B)** relative electrolyte leakage; **(C)** chlorophyll content (SPAD); **(D)** photochemical efficiency; **(E)** proline content; **(F)** Na^+^ content; **(G)** K^+^ content; **(H)** MDA content; **(I)** superoxide dismutase; **(J)** GSH content.

Under the treatment of 300 mM NaCl, seedlings of smooth bromegrass gradually withered from 12 h to 48 h (Supplementary Figure S1). Compared with the control group (CK), the relative water content gradually decreased under NaCl treatment, while the relative conductivity gradually increased and reached the peak at 48 h (Figures 1A,B). No significant difference in the chlorophyll SPAD value was found for the treatment group when compared with the control group, but the photochemical efficiency was reduced by 10.98% after 48-h treatment compared with the CK (Figures 1C,D). Proline and Na^+^ contents increased during treatment time (Figures 1E,F), while the K^+^ content increased slightly at 12 h and decreased gradually from 36 h to 48 h (Figure 1G).

The malondialdehyde content can be used as an indicator of membrane lipid peroxidation. The malondialdehyde level in leaves of smooth bromegrass was considerably elevated by 37.19% at 48 h in the NaCl treatment group compared to the control (Figure 1H). In order to investigate the antioxidant defense system of smooth bromegrass cells under NaCl treatment, activities of key enzymes such as superoxide dismutase (SOD) and reduced glutathione (GSH) were tested in both control and treatment groups. It showed that SOD activity reached its peak after a 12-h treatment and started to decrease from 24 h to 48 h (Figure 1I). Unlike SOD, the GSH content increased considerably to a maximum at 24 h and then decreased from 36 h to 48 h (Figure 1J).

### Statistical Analysis of Transcriptome Sequencing Data

The leaves and roots of smooth bromegrass treated with 300 mM NaCl at five time points were separately sampled and subjected to NGS sequencing. In total, about 226.11 G clean reads were generated by Illumina sequencing (Supplementary Table S2). The PacBio RS II platform was utilized to create the Iso-Seq library and single-molecule sequencing. In total, 19.67 G polymerase read bases and 516,677 polymerase reads were obtained. It showed that the final insert subread is 19.06 G, and the number of final insert subreads is 8,354,897 (Table 1).

**Table 1:** Full-length transcriptome sequencing results.

**Library**	**Bases(G)**	**Number**	**Mean length**	**N50**
Polymerase	19.67	516677	38,079	64,895
Subreads	19.06	8354897	2,282	2,730

In total, 369,646 full-length (FL) reads, 41,968 non-full-length (NFL) reads, and 355,836 full-length non-chimeric (FLNC) reads (Supplementary Table S3) were obtained. To eliminate redundancy, we used the ICE algorithm to cluster FLNC sequences with the same transcript, and a total of 202,837 consensus numbers were obtained. The length of the polished consensus sequence for each sample and the length distribution map are shown in Supplementary Figures S2A,B. In order to test the repeatability between samples, both principal component analysis and Pearson correlation were performed for a total of 30 samples. The principal component analysis showed the samples for L0 and R0 from leaves and roots were grouped separately, which was far away from the samples treated with salt (Supplementary Figure S3). Correlation analysis showed the Pearson correlation coefficient between samples of the same treatment was greater than 0.8 (Supplementary Figure S4), indicating that sampling was relatively accurate with small error, and the data analyzed later are also relatively reliable. Furthermore, the consensus sequences obtained from the subreads yielded 413,135 circular consensus sequences (CCS), and the average number of CCS was 17. FLNC reads accounts for 86.13% of the CCS (Supplementary Table S5).

**Figure 2:**
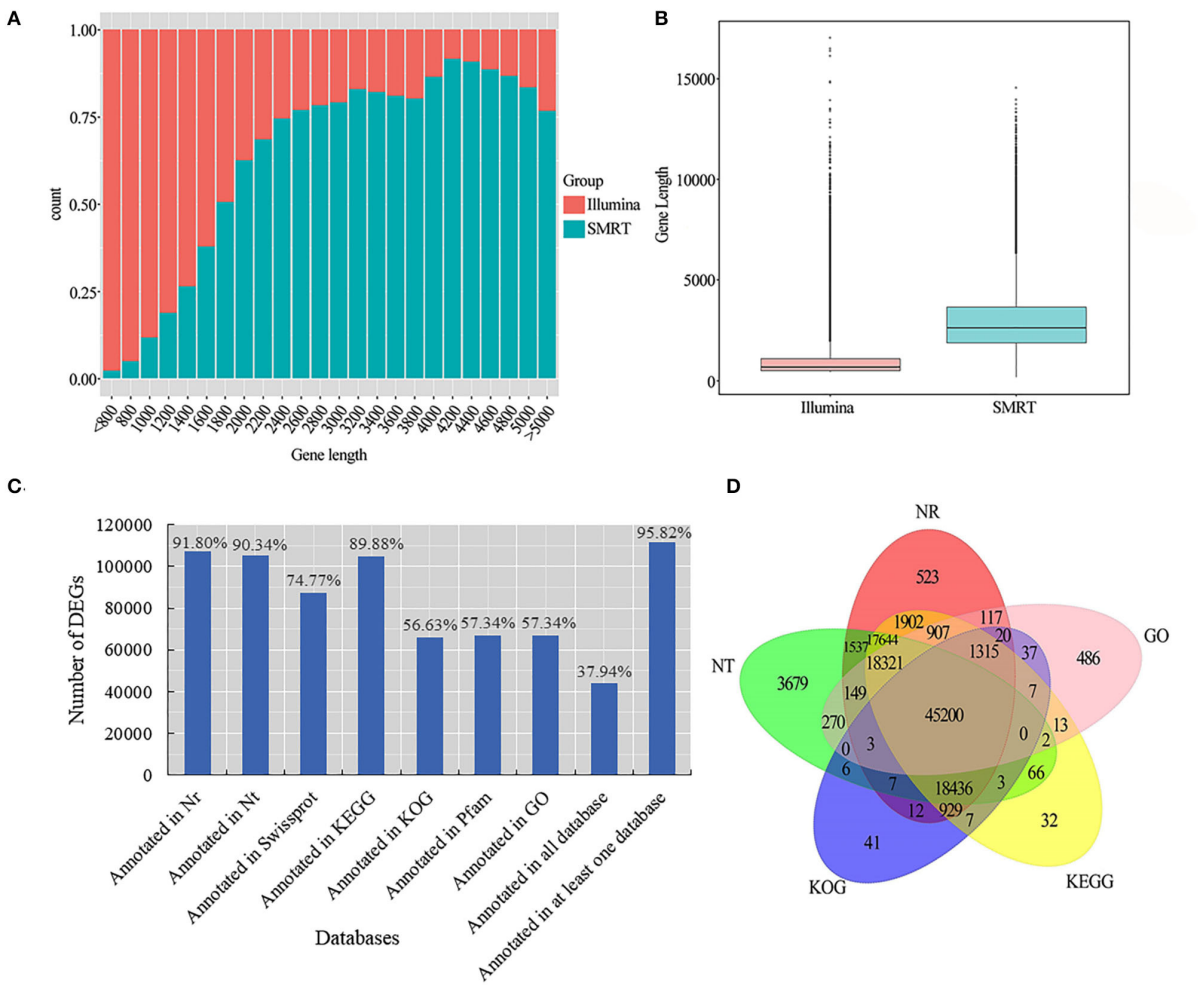
Unigene length distribution map and gene function annotation Venn diagram. **(A)** Illumina and SMRT measured the gene count distribution map. The abscissa is the length (nt) of the predicted gene; the ordinate is the number of transcripts reads of the gene; **(B)** Illumina and SMRT measured gene length distribution box diagram; **(C)** seven database annotation results statistical graph; **(D)** gene function annotation Venn diagram.

We used LoRDEC software (Leena and Eric, 2014) to correct and analyze the Illumina data and SMRT PacBio data, and CD-HIT software (Fu et al., 2012) to remove redundant and similar sequences, and finally, we obtained 202,837 transcripts (Table 2). Among all these transcripts, only 1.66% of them were >500 bp, 37.77% of them were 2-3kb, and 31.57% of them were longer than 3kb (Table 2). Our results confirmed that SMRT sequencing provides a large number of full-length and high-quality transcripts and that using Illumina data to correct low-quality SMRT reads improved the accuracy of long reads of PacBio (Figure 2A). The average length of genes detected by SMRT is more than that detected by Illumina. Finally, 116,578 unigenes were obtained after the removal of redundant sequences, and 1.11% of the retrieved unigenes were >500 bp, whereas 39.10% of them were longer than 3 kb (Table 2, Figure 2B).

**Table 2:** Statistical table of length frequency distribution before and after transcript de-redundancy.

**Transcripts length**					
**interval**	** <500bp**	**500-1kbp**	**1k-2kbp**	**2k-3kbp**	**>3kbp**	**Total**
Number of transcripts	3,362	11,784	59,214	64,439	64,038	202,837
Number of Genes	1,295	6,639	25,736	37,338	45,570	116,578

### Analysis of Gene Function Annotation

For more accurate gene analysis, we used ANGEL software (Shimizu et al., 2006) to predict CDS regions and obtained the distribution map of CDS (Supplementary Figure S3A). Unigenes were used for gene function annotation, with the following databases: Nr, Nt, Pfam, KOG/COG, SWISS-PROT, KEGG, and GO. In total, 66,847 (57.34%) unigenes had their annotation based on the GO database, and 104,784 (89.88%) and 107,022 (91.80%) unigenes based on KEGG and Nr databases, respectively (Figure 2C; Supplementary Table S4). Based on the plant species in the Nr database, a large number of unigenes were annotated to *Hordeum vulgare*, indicating that smooth bromegrass is closely related to *Hordeum vulgare* (Supplementary Figure S3B). Five major databases (viz., Nr, GO, KEGG, KOG, and Nt) jointly annotated 45,200 shared unigenes, and 523 were solely found in the NR database, 486 in the GO database, 32 in KEGG, 41 in KOG, and 3,679 in NT (Figure 2D).

### Analysis of Differentially Expressed Genes

**Figure 3:**
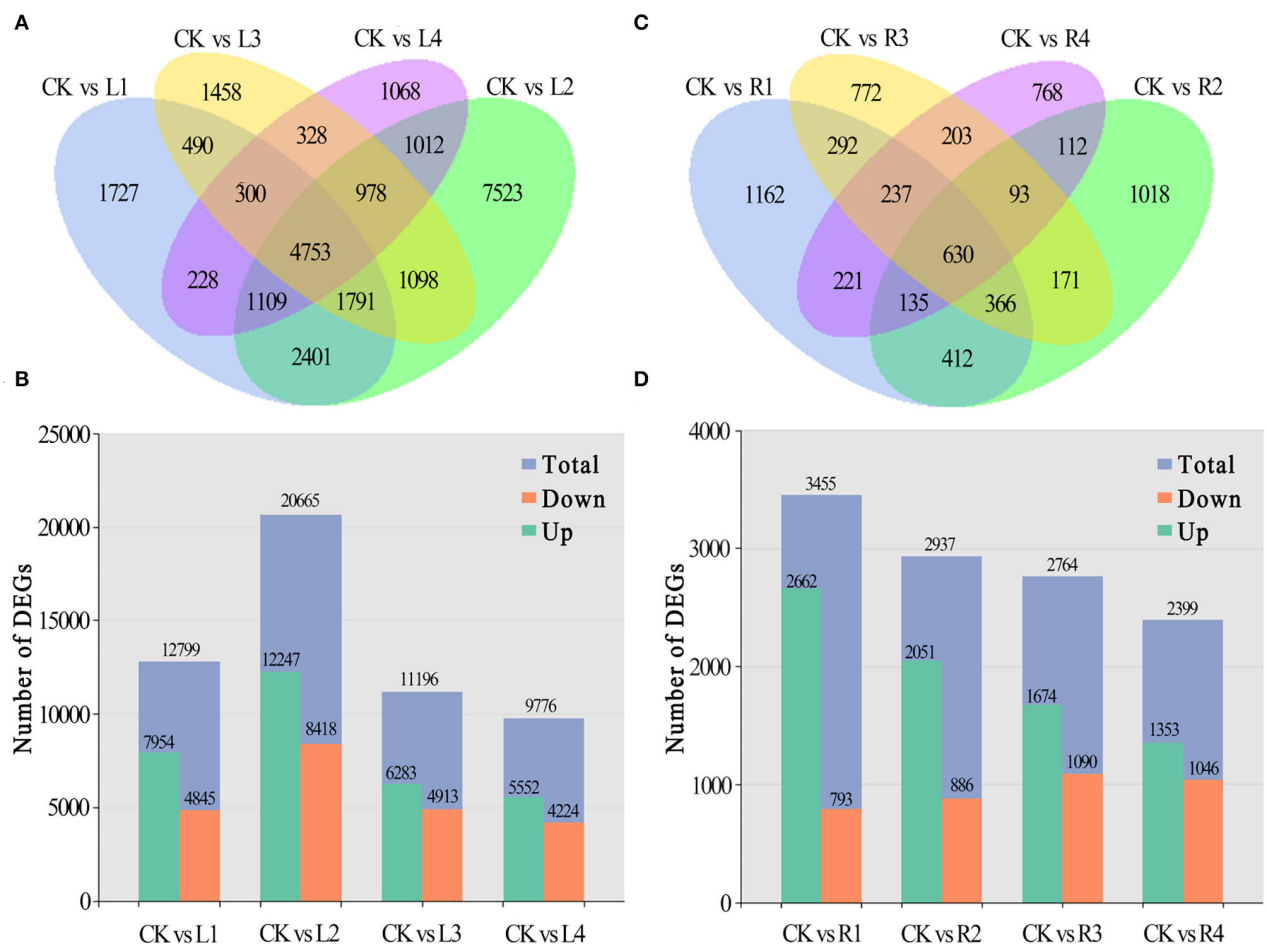
Distribution of DEGs in different periods. **(A,B)** Numbers of DEGs in leaves and roots of smooth bromegrass under different stress times compared with the control; **(C,D)** Summary of the numbers of up- and downregulated DEGs in leaves and roots of smooth bromegrass.

Compared with the control, 1,727 unigenes were specifically expressed in leaves under NaCl treatment at 12 h (L1), 7,523 at 24 h (L2), 1,458 at 36 h (L3), and 1,068 at 48 h (L4), and 4,753 of them were shared by these four comparison pairs (Figure 3A). Among differentially expressed genes, 7,954, 12,247, 6,283, and 5,552 unigenes were upregulated, and 4,845, 8,418, 4,913, and 4,224 unigenes were downregulated for the four comparison pairs, respectively (Figure 3B).

In roots, 1,162 unigenes were specifically expressed at the R1 stage under NaCl treatment in comparison with the control group, 1,018 at the R2 stage, 772 at the R3 stage, and 768 at the R4 stage, and a total of 630 unigenes were shared by the four comparison pairs (Figure 3C). Meanwhile, 3,455, 2,937, 2,764, and 2,399 unigenes were found to be differentially expressed in roots of smooth bromegrass at four different periods in comparison with the control group. Among them, 2,662, 2,051, 1,674, and 1,353 unigenes were upregulated, and 793, 886, 1,090, and 1,046 unigenes were downregulated at the four stages, respectively (Figure 3D).

### Analysis of GO Annotation

**Figure 4:**
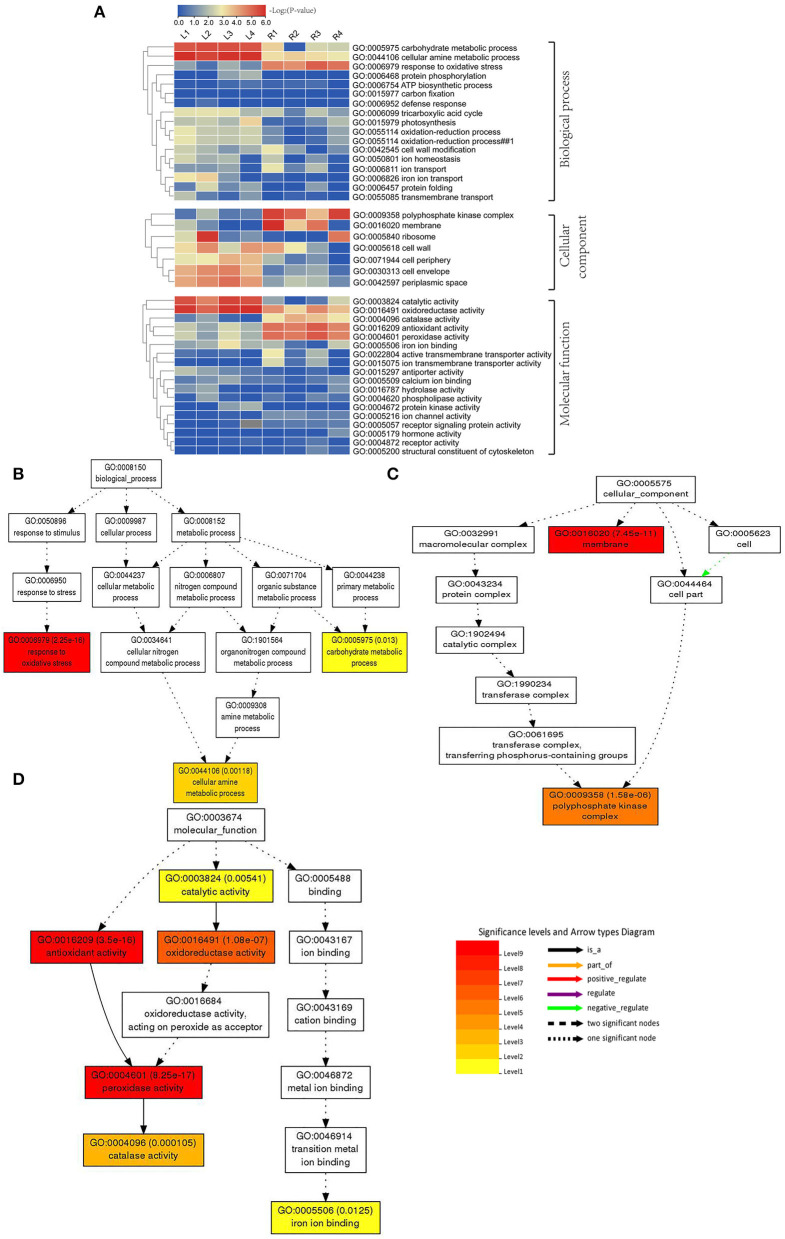
GO enrichment results of DEGs after NaCl treatment. **(A)** GO annotation results of DEGs; **(B**–**D)** GO term for DEGs that respond to salt stress. Each box represents a GO term; the depth of the color represents the degree of enrichment, and the darker the color, the higher the degree of enrichment.

The GO database was used for the annotation of DEGs between the control and treatment groups. The heat map for GO of DEGs was generated based on the calculated -log_2_(*p*-value), and a total of 17 biological processes were identified (Supplementary Table S5). Among them, the carbohydrate metabolism process, cellular amine metabolism process, and response to oxidative stress process showed higher expression levels in each stage under salt stress (Figure 4A). These data were also analyzed by using a direct acyclic graph (DAG) tree, resulting in the identification of response to oxidative stress, carbohydrate metabolic process, cellular amine metabolic process, and other important processes (Figure 4B).

Furthermore, we found seven processes related to cell components (Supplementary Table S5), which were expressed differently in both leaves and roots. The DAG tree analysis also revealed important processes of the polyphosphate kinase complex and membrane (Figure 4C). By analyzing these data, we also found 18 processes related to molecular functions (Supplementary Table S5), and these processes varied in different stages under NaCl treatment. In particular, the catalytic activity process was significantly expressed in leaves of smooth bromegrass at all treatment time points (Figure 4A). The oxidoreductase activity process exhibited relatively high expression in various processing stages in both leaves and roots. In the process of peroxidase activity, catalase activity and antioxidant activity showed higher expression in roots. Moreover, several important molecular functional processes were also detected by using DAG analysis, such as catalytic activity, antioxidant activity, oxidoreductase activity, peroxidase activity, catalase activity, and iron ion binding (Figure 4D).

### Analysis of KEGG Pathway

**Figure 5:**
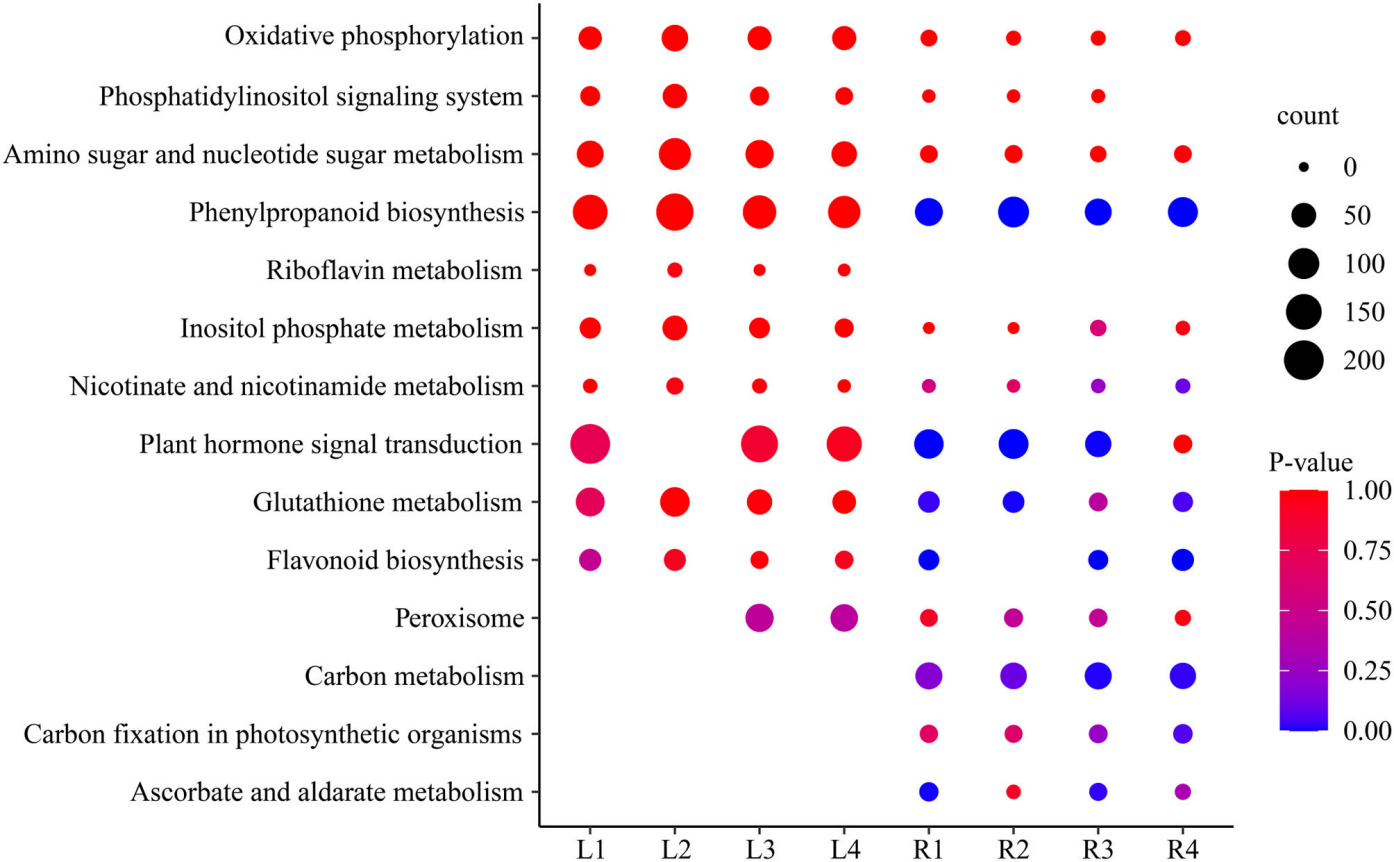
Scatter plot of the enriched KEGG pathway of DEGs under NaCl stress.

Analysis of the KEGG metabolic pathway was performed with DEGs between the control group and the four treatment groups in both leaves and roots, and 14 important pathways were significantly enriched (Supplementary Table S6), including ascorbate and aldarate metabolism, oxidative phosphorylation, glutathione metabolism, plant hormone signal transduction, phenylpropanoid biosynthesis, and starch and sucrose metabolism (Figure 5). These pathways were affected in different time periods under salt stress. The phenylpropanoid biosynthesis pathway, oxidative phosphorylation, phosphatidylinositol signaling system, and sugar metabolism were highly expressed, with more genes in leaves than in roots. Interestingly, the pathways of riboflavin metabolism only occurred in leaves but not in roots, while the carbon metabolism pathway, carbon fixation in photosynthetic organisms, and ascorbate and aldarate metabolism only occurred in roots but not in leaves, indicating they are tissue-specific pathways in smooth bromegrass (Figure 5).

### Analyses of Gene Expression Levels Involved in Specific Pathways

In this study, we also analyzed ABA signaling pathway genes and identified 56 genes encoding signal receptor sensors related to NaCl stress through DEGs annotation analysis (Supplementary Figure S6A and Supplementary Table S7). In addition, 3 *RLKs*, 8 *CDPKs*, 20 *CIPKs*, 15 *CMLs*, and 10 *MAPKs* in the signal sensor pathway were also identified (Supplementary Figure S6B and Supplementary Table S8).

**Figure 6:**
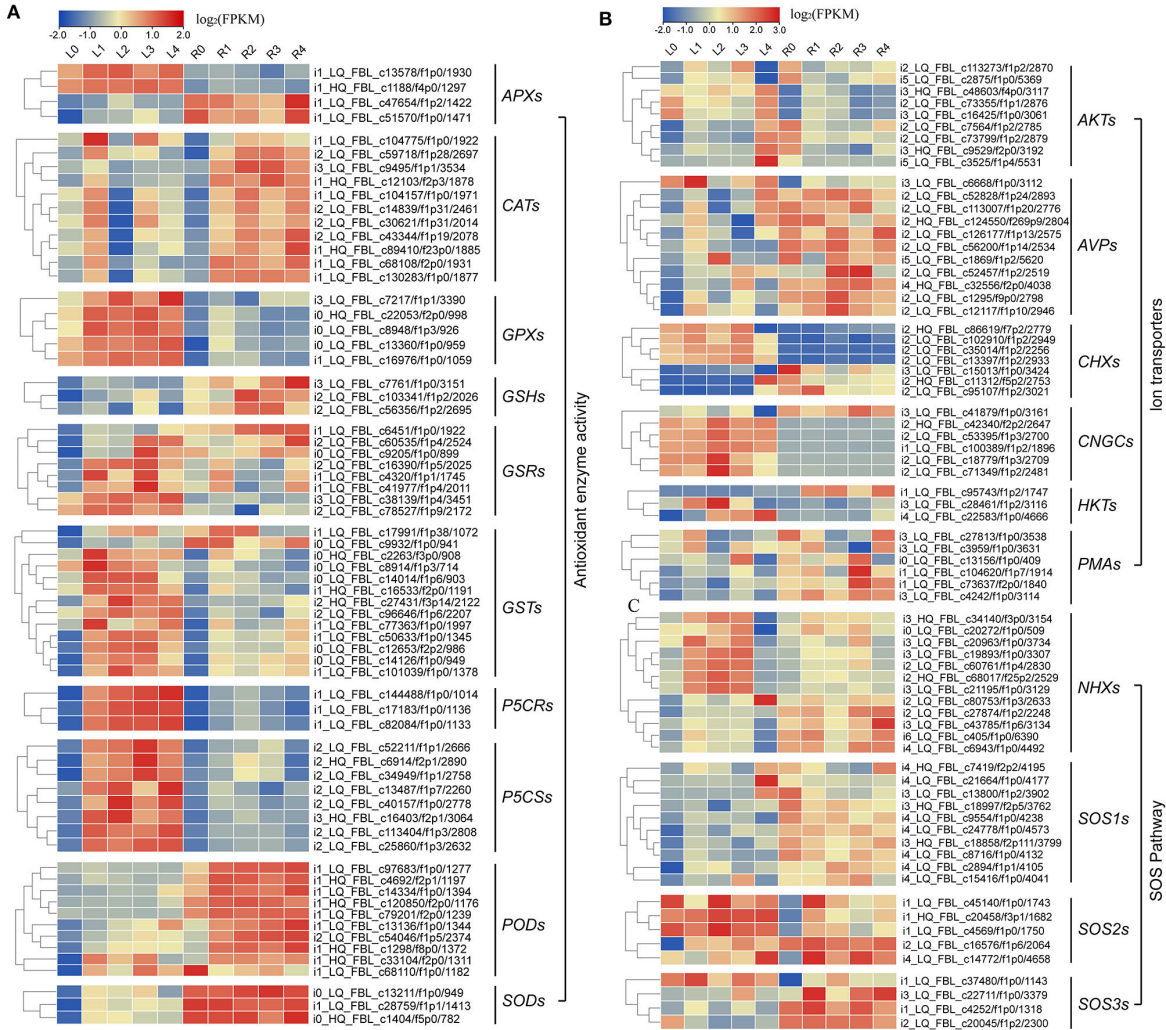
Heat map plot of the expression levels of the DEGs involved in antioxidative, SOS, and ion transporter pathway under NaCl stress. **(A)** Heat map plot of the antioxidative defense-related DEGs by NaCl stress; **(B,C)** heat map plot of the DEGs of SOS pathway and ion transporters by NaCl stress.

Meanwhile, we also found that most of the DEGs were associated with antioxidant enzymes (*SODs, PODs, GPXs, CATs*, and *APXs*), non-enzymatic antioxidants (*GSTs, GSHs*, and *GSRs*), and proline synthase (*P5CSs*, and *P5CRs*), and their corresponding coding genes were all regulated by NaCl stress at various levels (Figure 6A; Supplementary Table S9). We found that the expression levels of 9 *AKTs*, 11 *AVPs*, 7 *CHXs*, 6 *CNGCs*, 3 *HKTs*, 3 *KATs*, and 6 *PMAs* genes were also affected under NaCl treatment in both leaves and roots (Figure 6B; Supplementary Table S10). Furthermore, we identified 12 *NHX-*, 10 SOS1-, 5 SOS2-, and 4 SOS3-related genes (Figure 6C, Supplementary Table S11), and these genes were upregulated after salt stress, indicating that Na^+^ regulatory mechanism in smooth bromegrass was activated.

### Identification of Salt-Responsive Transcription Factor Genes

**Figure 7:**
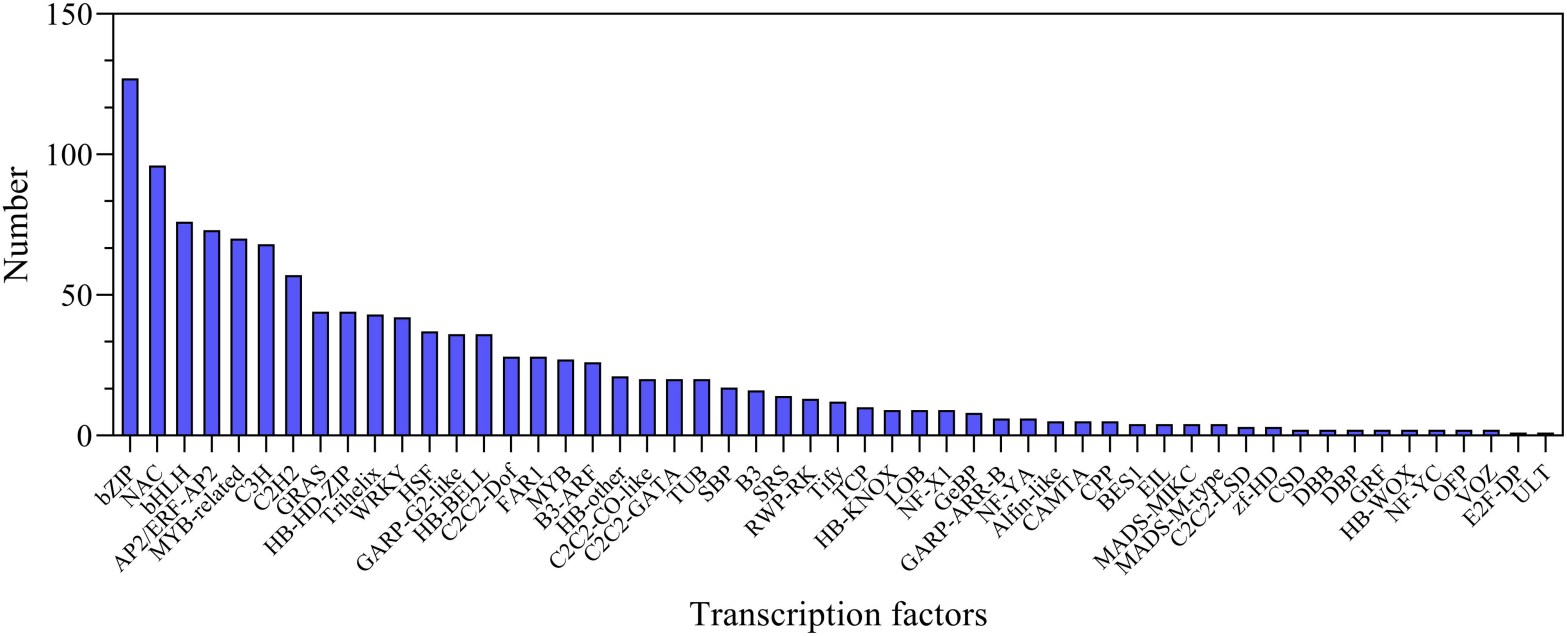
Statistics of transcription factors under NaCl stress.

In this study, a total of 1,223 transcription factor genes were identified and classified into 53 families. Among them, the bZIP family is the largest family with 127 genes, followed by 96 *NAC*-, 76 *bHLH*-, 73 *AP2/ERF*-, and 70 *MYB-*related genes (Figure 7; Supplementary Table S12). In order to investigate the expression of these TF genes, they were all analyzed at all the treatment time points. It was found that the expression levels of many transcription factor genes were highly upregulated at different time points after salt stress, including several main TF family genes *AP2/ERF, bHLH, bZIP, GRAS, MYB*, and *WRKY* (Supplementary Figure S7). In particular, many of them showed distinct expression profiles between leaves and roots (Supplementary Figure S7). The dynamic changes in the expression level of these TF genes implied their important roles in regulating plant salt tolerance.

### Validation of NaCl Stress-Related Genes by RT-qPCR

To validate the transcriptome data, 10 DEGs were randomly selected, and their expression levels were analyzed in the control and treatment groups by RT-qPCR (Supplementary Figure S8A), with *actin* gene as an internal reference. These genes included the putative peroxide gene, MYB39, and CYP73A2 (Supplementary Figure S8A). By comparing and analyzing these results with the RNA-Seq data (Supplementary Figure S8B), it was found that the expression of these 10 selected genes showed similar expression profiles in the leaves and roots at four time points (Supplementary Figure S8A). Further analyses showed that RT-qPCR and RNA-Seq data were correlated linearly either in leaves or in roots at 12 h, 24 h, 36 h, and 48 h (Supplementary Figure S8B–E), indicating RNA-Seq data were reliable for further gene screening and expression level comparison.

## Discussion

**Figure 8:**
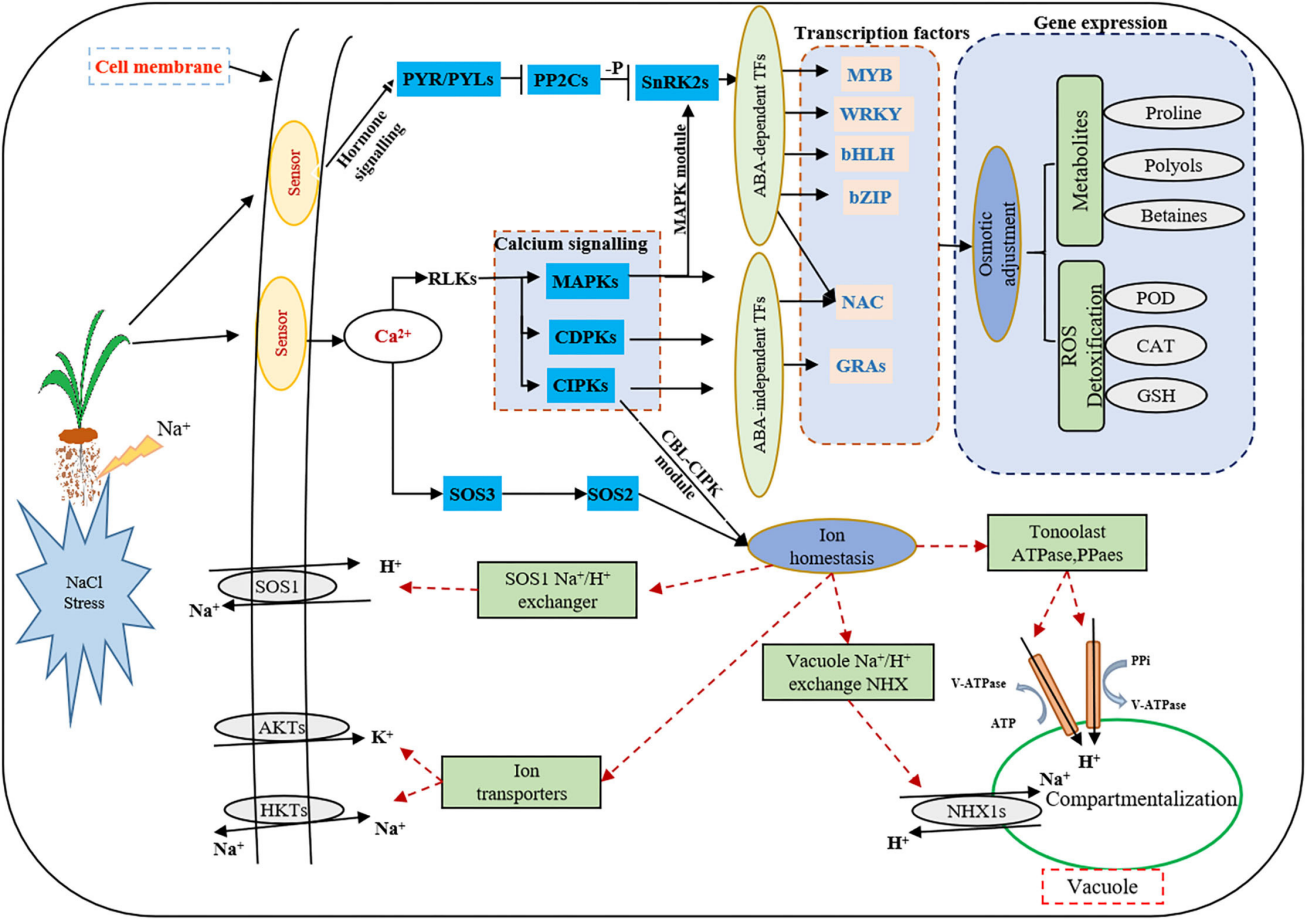
Model describing the signal transduction pathways involved in the acquisition of salt tolerance in smooth bromegrass. Red dashed lines indicate proposed regulation, solid arrowheads indicate activation, and vertical solid lines indicate suppression.

At present, the third-generation sequencing technology (e.g., PacBio SMRT) has greatly facilitated the *de novo* assembly of transcriptomes of many higher organisms and has helped to overcome the problems of short splicing and incomplete information for the species without reference genomes (Cheng et al., 2017; Chao et al., 2019; Kan et al., 2020; Shen et al., 2021). Meanwhile, second-generation sequencing (e.g., Illumina platform) has been widely used to obtain more comprehensive annotation information of many plant species (Yang et al., 2018; Luo et al., 2019). In this study, for the first time, the Illumina platform was used to study the transcriptome changes of global genes in response to salt stress in the leaves and roots of smooth bromegrass, and the reference sequence was obtained from its full-length transcript database using PacBio SMRT sequencing technology. After screening, we acquired 19.67 G polymerase read bases (Table 1) and 116,578 distinct genes in 30 sample libraries (Table 2). Of these genes, 95.82% of DEGs were annotated in at least one database (Figure 3C), and five major databases (viz., Nr, GO, KEGG, KOG, and Nt) jointly annotated 45,200 unigenes (Figure 3D), which is higher than *Elymus sibiricus* (79.81%) (Zhou et al., 2016); however, it is lower than alfalfa (99%) (Luo et al., 2019). Compared with the control, 12,799, 20,665,11,196, and 9,776 DEGs were specifically expressed in leaves under NaCl treatment at 12 h, 24 h, 36 h, and 48 h, respectively (Figure 3B), and in roots, 3,455, 2,937, 2764, and 2,399 DEGs were identified in four different periods (Figure 3D). The candidate genes were analyzed in the control and treatment groups by RT-qPCR (Supplementary Figure S8A), which were consistent with the RNA-Seq data, implying our sequencing data were reliable (Supplementary Figure S8). Our study provided valuable and nearly complete sequence information on smooth bromegrass. Furthermore, we depicted a global molecular mechanism model of salt response in smooth bromegrass (Figure 8), with all these transcriptome information.

Under salt stress conditions, plants produced a large number of genes related to osmotic regulation, which regulate the accumulation of primary metabolites in plants such as proline, betaine, and sugar alcohol to maintain cell osmotic potential and improve their own salt tolerance (Xiong et al., 2017; Singh et al., 2018; Gao et al., 2019). Our studies showed that proline and soluble sugar contents increased in smooth bromegrass leaves under salt treatment (Figure 1), which was consistent with other studies in *Arabidopsis thaliana* (Yang et al., 2018), *Medicago sativa* (Luo et al., 2019), *Sorghum bicolor* (Cui et al., 2018), *Arundo donax* (Sicilia et al., 2020), and *Zea mays* (Kakumanu et al., 2012), indicating that plants share similar salt tolerance mechanisms. At the molecular level, the GO categories “response to oxidative stress,” “ion transport,” “catalytic activity,” “antioxidant activity,” and “hormone activity”, and the KEGG pathway categories “plant hormone signal transduction,” “phenylpropanoid biosynthesis,” and “flavonoid biosynthesis” were significantly enriched under salt stress (Figures 4, 5), which were consistent with rice (*Oryza sativa*) (Rabbani et al., 2003), maize (*Zea mays*) (Sun et al., 2015), and *Sophora alopecuroides* (Zhu et al., 2021). These results implied secondary metabolic processes and antioxidant systems are activated through secondary metabolites (phenylpropane and flavonoids), hormones, ion transport (Na^+^ and K^+^), antioxidant enzymes (SOD and GSH), and second messengers (such as ROS and Ca^2+^) in response to salt stress. Consistently, our physiological work also revealed that SOD and GSH contents increased at the physiological level by NaCl stress (Figures 1I,J), and increased levels of Na^+^ and K^+^ contents were consistent with the ion transport pathway (Figures 1F,G). The physiological regulation mechanism of salt stress usually includes osmotic regulation, ion transmembrane transport, ion compartmentalization, and active oxygen scavenging. Plant hormones and transcription factors also respond to salt stress (Zhu, 2016; Geng et al., 2020; Luo et al., 2020), which enabled smooth bromegrass to respond to salt stress and further adapt to changes in the external environment by ROS accumulation.

Under NaCl stress, the osmotic stress caused by the increase in the Na^+^ concentration in the rhizosphere of plants reduces the water potential. On the other hand, this ion stress induces a nutritional imbalance in plants (Huang et al., 2012; Al-Farsi et al., 2020). The active oxygen signal pathway is activated in response to osmotic stress, and excessive ROS causes serious damage to plant cell membranes, proteins, nucleic acids, etc. (Yun et al., 2011; Zhang et al., 2016). Plants produce redox-sensitive TFs and other molecular sensors to perceive stress signals, thus increasing the ROS concentration and activating ROS defense/metabolic pathways for ROS clearance (Chawla et al., 2013; Fidalgo et al., 2015; Mahmoud et al., 2020). Physiological studies on smooth bromegrass leaves revealed that NaCl produced oxidative damage, resulting in a substantial reduction in the chlorophyll content, an elevation in relative electrical conductivity, and a reduction in the relative water content (Figure 1). Smooth bromegrass activates the antioxidant defense system by adjusting the activity of antioxidants (POD, CAT, and SOD) and the content of osmotic regulators (PRO) (Supplementary Table S9). The same transcriptome data also revealed that NaCl stress activates the antioxidant defense system at the molecular level. When exposed to NaCl treatment GO-type oxidoreductase activity and KEGG pathways peroxisome and glutathione metabolism, as well as hormone signal transduction, were all enriched, as illustrated in Figures 4, 5. In addition, most of the DEGs associated with antioxidant enzymes (*SODs, PODs, GPXs, CATs*, and *APXs*), non-enzymatic antioxidants (*GSTs, GSHs*, and *GSRs*), and proline synthase (*P5CSs* and *P5CRs*) were all regulated by NaCl stress (Supplementary Table S9). These physiological indicators are consistent with the results of the transcriptome data, which strongly indicate the important role of these DEGs in responding to osmotic stress and therefore protecting smooth bromegrass from ROS damage.

Plants respond to external stress by activating signal pathways, including the ROS signal pathway, the MAPK cascade signal system, the ABA signal response pathway, and Ca^2+^-dependent proteins (Wang et al., 2016b; Danquah et al., 2014; Steinhorst and Kudla, 2019). Osmotic stress signals are sensed by protein receptors on the cell membrane. These stress signal receptors can quickly sense changes in the external environment. Subsequently, many second messengers such as Ca^2+^, inositol phosphate, ROS, and ABA signals will be rapidly produced in the cytoplasm to further decode and amplify the salt stress signal (Mehlmer et al., 2010; Zhu, 2016; Cao et al., 2020). The second messenger stimulates downstream signals through protein phosphorylation cascade changes, such as CDPKs, CBLs, CIPKs, CMLs, and MAPKs, linking the perception of external stimuli with cellular responses (Danquah et al., 2014; Bakshi et al., 2019). In this study, we identified a number of genes encoding signal receptor sensors related to NaCl stress through DEG annotation analysis (Supplementary Figure S4B), including *CDPKs, CIPKs, CMLs*, and *MAPKs* (Supplementary Table S8). Since roots are directly exposed to salt stress, the gene expression of smooth bromegrass exhibits distinct expression profiles in leaves and roots.

The characteristic of the CBL-CIPK regulatory network provides a great contribution to the efficient transduction of Ca^2+^ signals and also plays an important role in regulating the response of plants to salt stress (Thoday-Kennedy et al., 2015; Liu et al., 2021). The CBL-CIPK module can interact with many ion transporters and regulate their activity, especially Na^+^/K^+^ transporters, which are essential for ion homeostasis (Pandey et al., 2015; Fan et al., 2016). Studies have reported that cyclic nucleotide-gated channels (CNGCs) can mediate the flow of Ca^2+^ into the cytoplasm under stress and maintain ion balance in the cell (Kugler et al., 2009; Jin et al., 2015). In many cases, these ion transporters interact with the SOS signaling network in a synergistic or antagonistic manner to maintain ion “balance” in plants under salt stress conditions. In our study, the GO enrichment of DEGs revealed genes involved in ion homeostasis, iron ion transport, ion channel activity, ion transmembrane transporter activity, and iron ion binding, as well as the ion transporter (Figure 4). Meanwhile, genes involved in this signaling pathway were identified, including *AKTs, AVPs, HKTs*, and *PMAs* (Figure 6B; Supplementary Table S10), indicating that these DEGs may directly or indirectly promote the tolerance mechanism of smooth bromegrass to salt stress. The SOS signal pathway is an important signal transduction pathway for plant salt tolerance, and it plays an important role in regulating Na^+^ and K^+^ homeostasis and salt tolerance (Deinlein et al., 2014; Steinhorst and Kudla, 2019). In total, 31 genes related to NHXs and SOS pathways were identified (Supplementary Table S11), and they were largely upregulated by salt stress (Figure 6B), indicating that Na^+^ regulatory mechanism was activated in smooth bromegrass, which was consistent with an increase in the Na^+^ content (Figure 1F).

ABA is an important plant hormone that plays a key role in regulating plant growth and resisting adversity stress. The ABA signaling pathway is also activated in the early stage of osmotic stress (Raghavendra et al., 2010; Komatsu et al., 2020). Osmotic stress causes plants to accumulate a significant quantity of active oxygen, and NADPH oxidase in the ABA pathway has a certain mitigating effect on alleviating osmotic stress (Zhao et al., 2020). In this study, 36 ABA signaling pathway-related genes were analyzed, including 5 *PYR/PYLs*, 19 *PP2Cs*, and 12 *SnRK2s* (Supplementary Figure S6A; Supplementary Table S7), and the expression level of these genes increased significantly after salt stress treatment in smooth bromegrass.

TFs are key components in the osmotic stress signal pathway, and they are involved in the signal perception of salt stress and the expression of downstream key genes in response to salt stress. They participate in a variety of biological processes and play an important role in regulating plant growth and stress response (Hartmann et al., 2015; Yang et al., 2020). The transcriptome of smooth bromegrass revealed that a number of different transcription factor families were affected by salt stress, including *bZIP, NAC, bHLH, WRKY, AP2/ERF, MYB*-related, *C3H, C2H2, GRAS*, and *MYB* (Figure 7), which was similar to those major TF families as in wheat under salt stress (Malik et al., 2020). These transcription factor genes have also been found in response to salt stress of *Arabidopsis thaliana* (Yang et al., 2018), *Caragana korshinskii* (Li et al., 2016), and *Cynodon dactylon* (Hu et al., 2015). Transcription factors such as *AP2/ERF, bHLH, bZIP, C2H2, C3H, GRAS, NAC, MYB-*related, and *WRKY* showed different expression patterns in leaves and roots after NaCl stress (Supplementary Figure S7), indicating that NaCl had different effects on the complex transcriptional regulation of smooth bromegrass adaption to salt stress.

## Data Availability Statement

The datasets presented in this study can be found in online repositories. All sequencing data of PacBio Iso-Seq and Illumina SGS RNA-Seq in this article can be obtained in NCBI SRA (http://www.ncbi.nlm.nih.gov/sra): SRR15634023 and SRR15633796-SRR15633815.

## Author Contributions

JS and QL designed this experiment, performed the experiments, and drafted the manuscript. JS, YC, and LZ analyzed experimental data and visualized it. BZ, YP, and YC revised the manuscript and directed the study. All authors have read and agreed to the published version of the manuscript.

## Funding

This research was funded by the China Agriculture Research System of MOF and MARA (CARS-34).

## Conflict of Interest

The authors declare that the research was conducted in the absence of any commercial or financial relationships that could be construed as a potential conflict of interest.

## Publisher's Note

All claims expressed in this article are solely those of the authors and do not necessarily represent those of their affiliated organizations, or those of the publisher, the editors and the reviewers. Any product that may be evaluated in this article, or claim that may be made by its manufacturer, is not guaranteed or endorsed by the publisher.
