# Supplementary material for: Full-Length Transcriptomics Reveals Complex Molecular Mechanism of Salt Tolerance in Bromus inermis L
Source: Front Plant Sci. 2022 Jun 9;13:917338. doi: 10.3389/fpls.2022.917338 (PMC9219601; doi:10.3389/fpls.2022.917338)
Supplement: Supplementary file 2 [file Data_Sheet_2.docx]

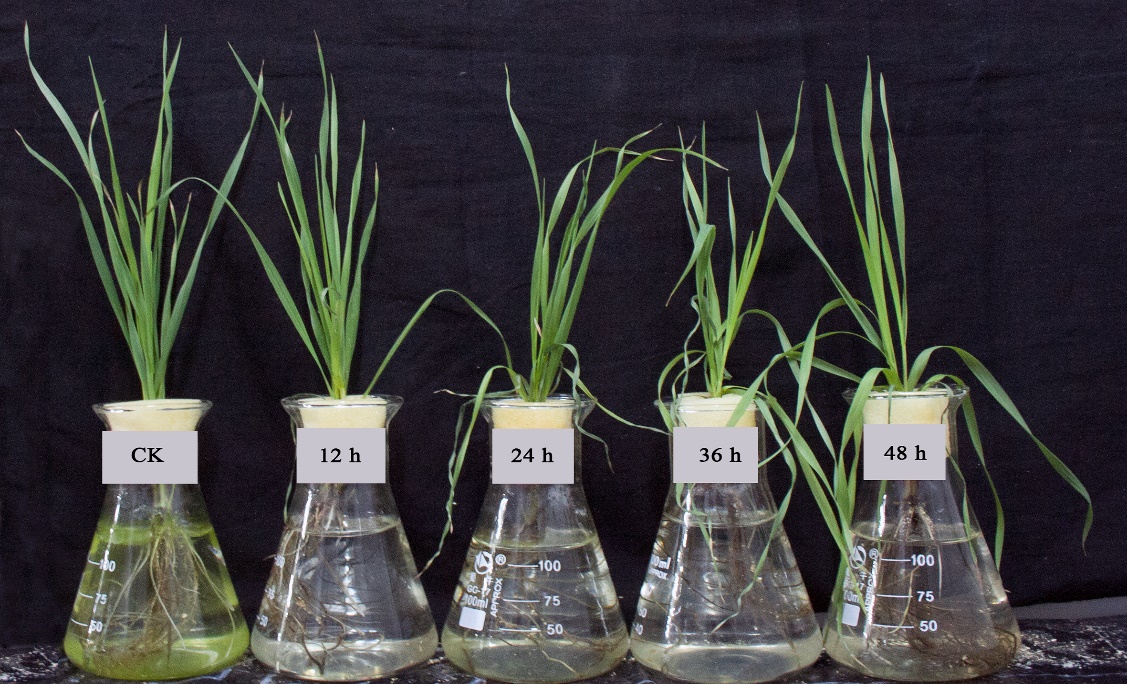


**Figure S1. Phenotypic changes in different stages of NaCl treatment (300 mM)**

Note: CK: Untreated; 12 h:salt treatment for 12 hours; 24 h: salt treatment for 24 hours; 36 h: salt treatment for 36 hours; 48 h: salt treatment for 48 hours.


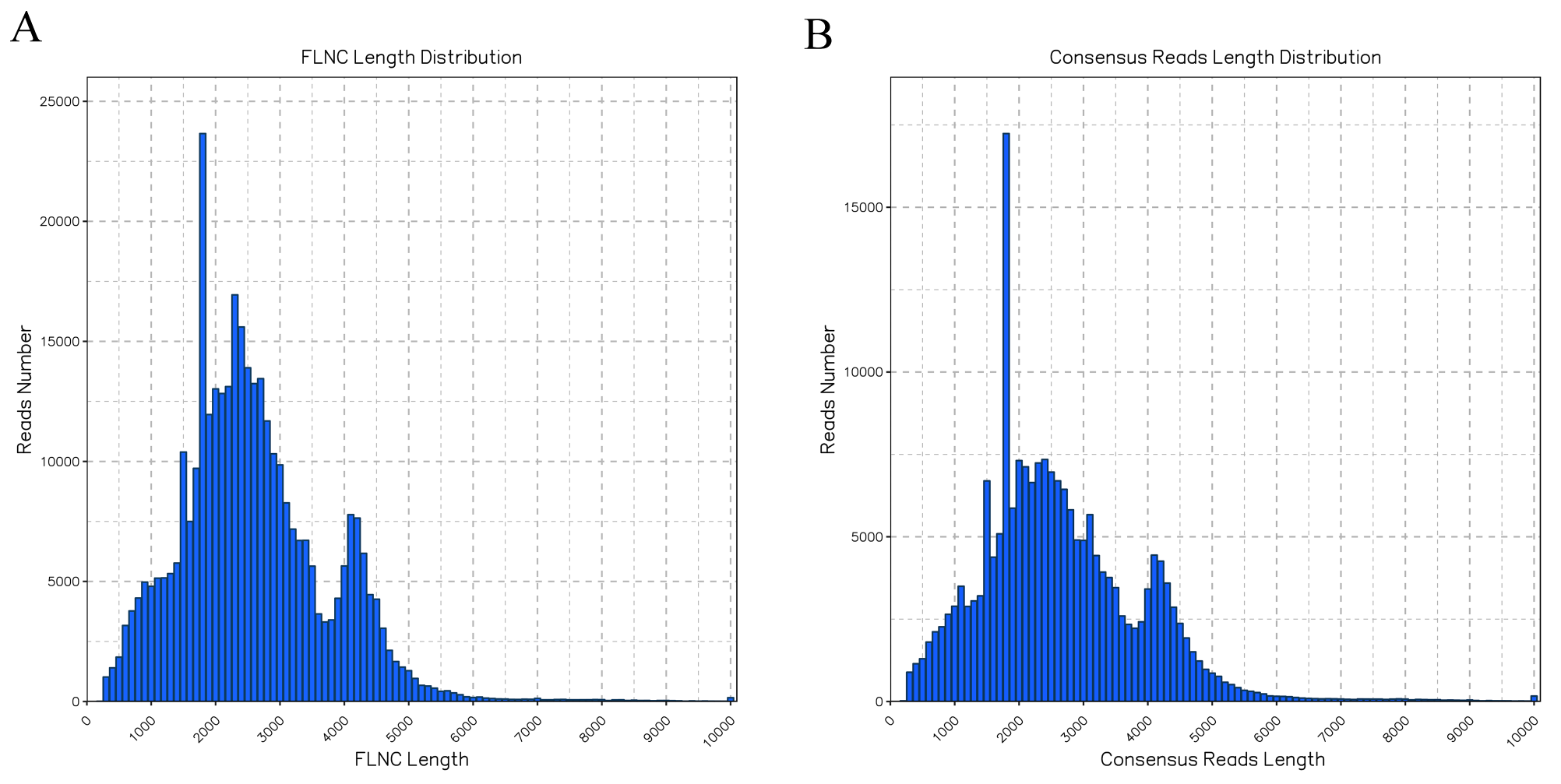


**Figure S2. FLNC length and consensus reads length distribution**

Note: a: FLNC length distribution map; b: Consensus reads length distribution map


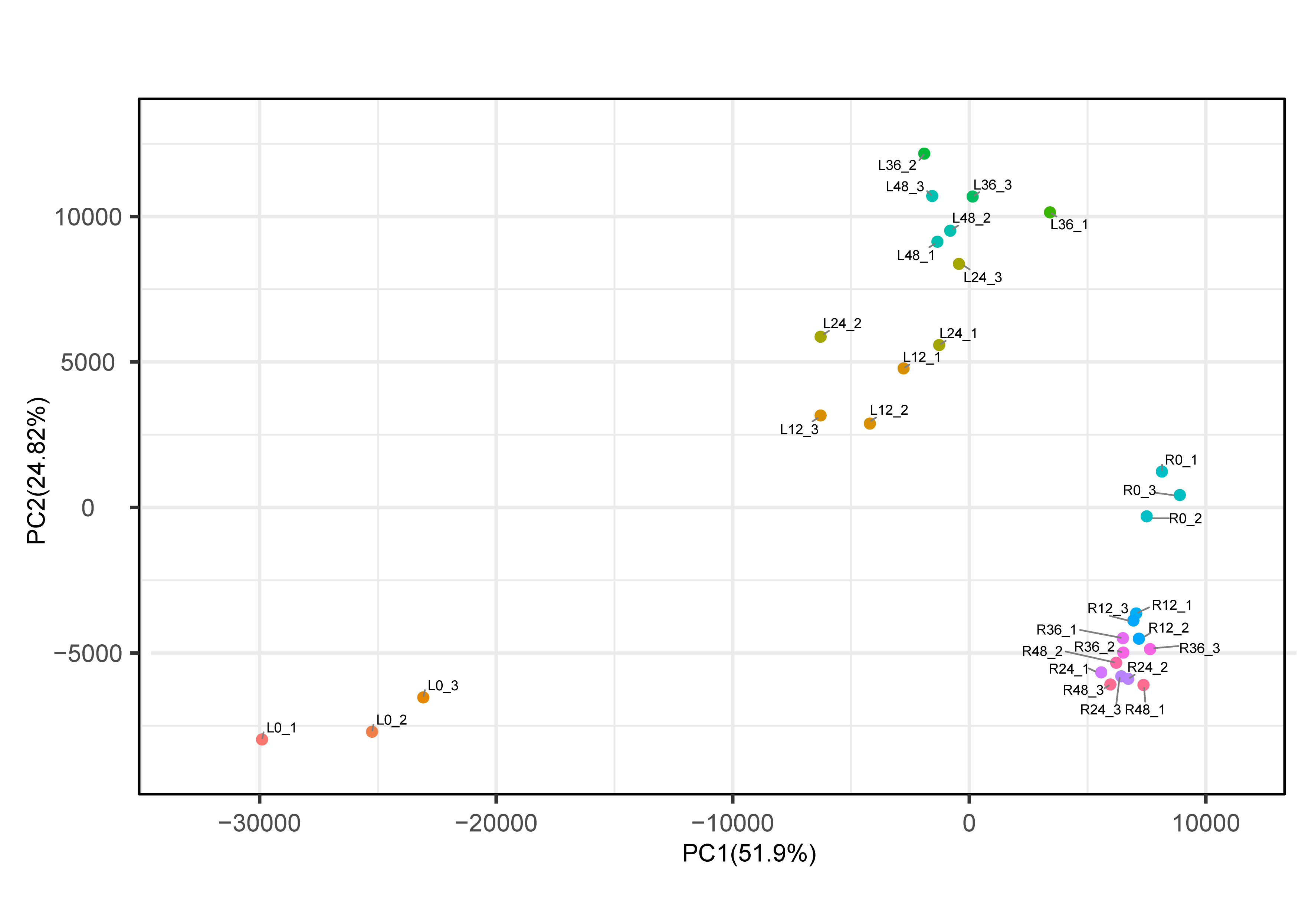


**Figure S3.** **PCA analyses of *Bromus inermis* under salt stress.**

**
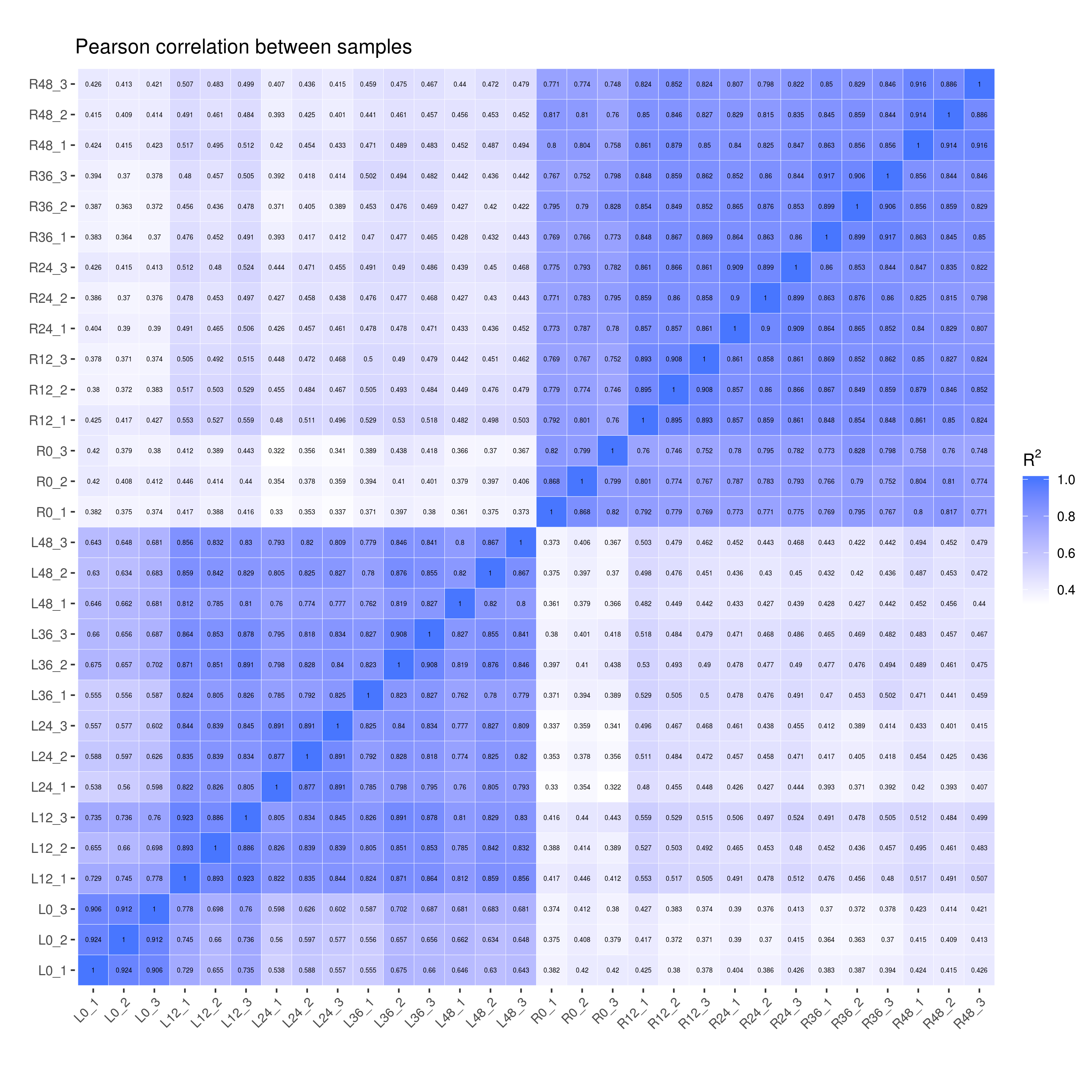
**

**Figure S4. Pearson correlation between samples.**


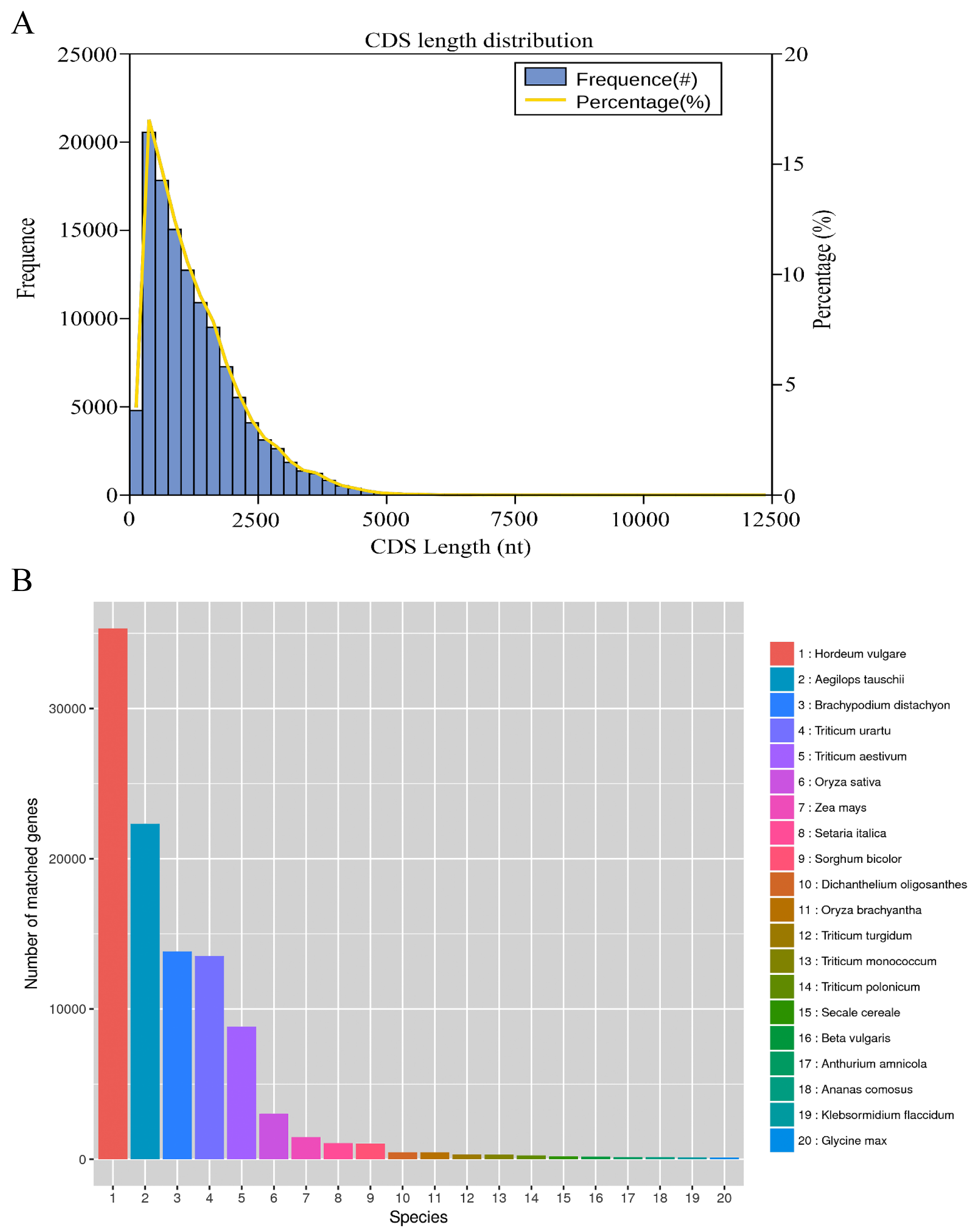


**Figure S5. The distribution map of the length of the CDS prediction results and the species annotation statistics of the NR database**

Notea: Distribution diagram of the length of CDS prediction results. b. The distribution map of the species annotation statistics of the NR database


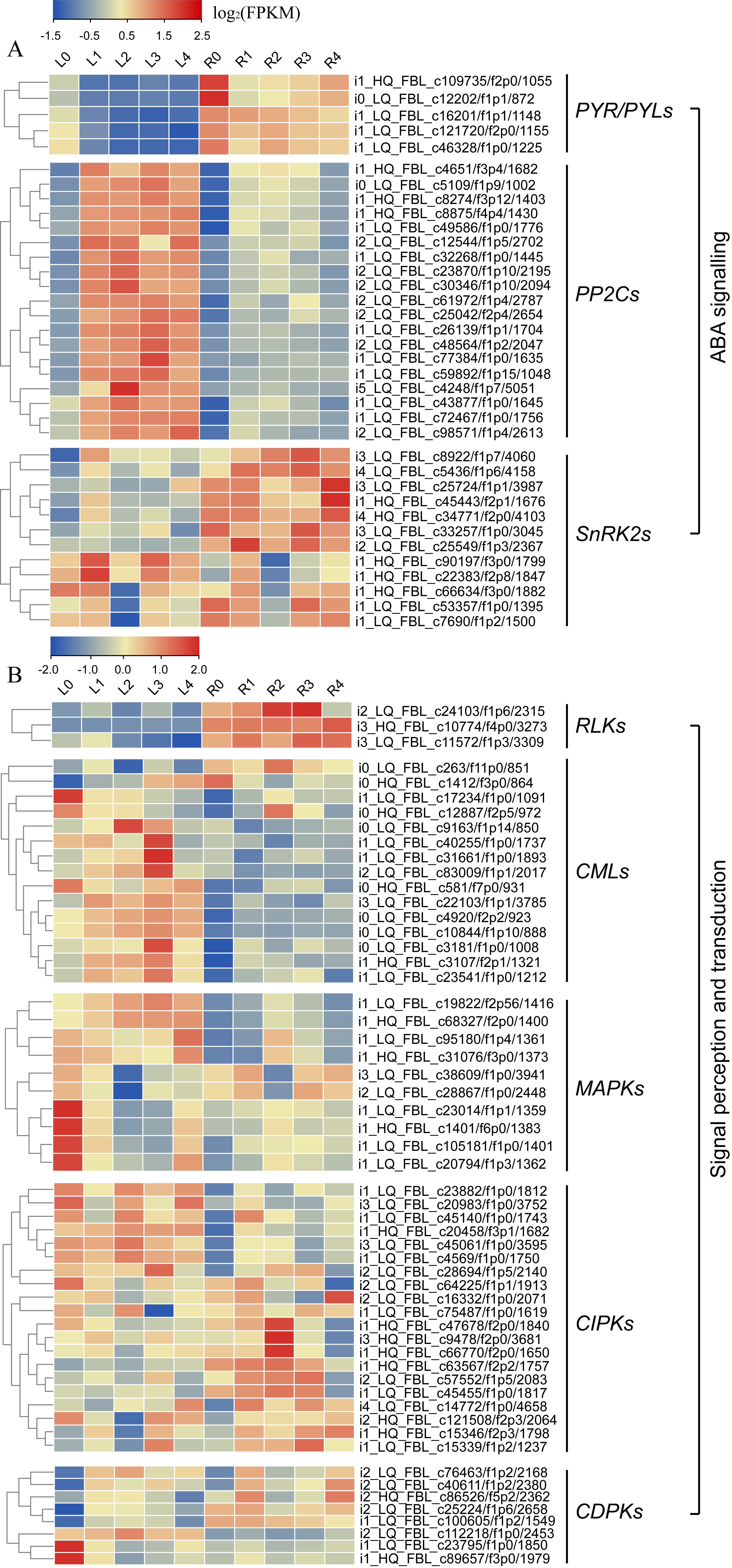


**Figure S6. Heatmap plot of the expression levels of the DEGs of Signal sensors-related and ABA signaling-related by NaCl stress**

Note: The gene expression is based on the z-scores of log_2_(FPKM) value. A. Heatmap plot of the expression levels of the ABA signalling related DEGs. B. Heatmap plot of the expression levels of the Signal Perception and transduction related DEGs. The red and blue indicate high and low expression levels of differential genes, respectively.


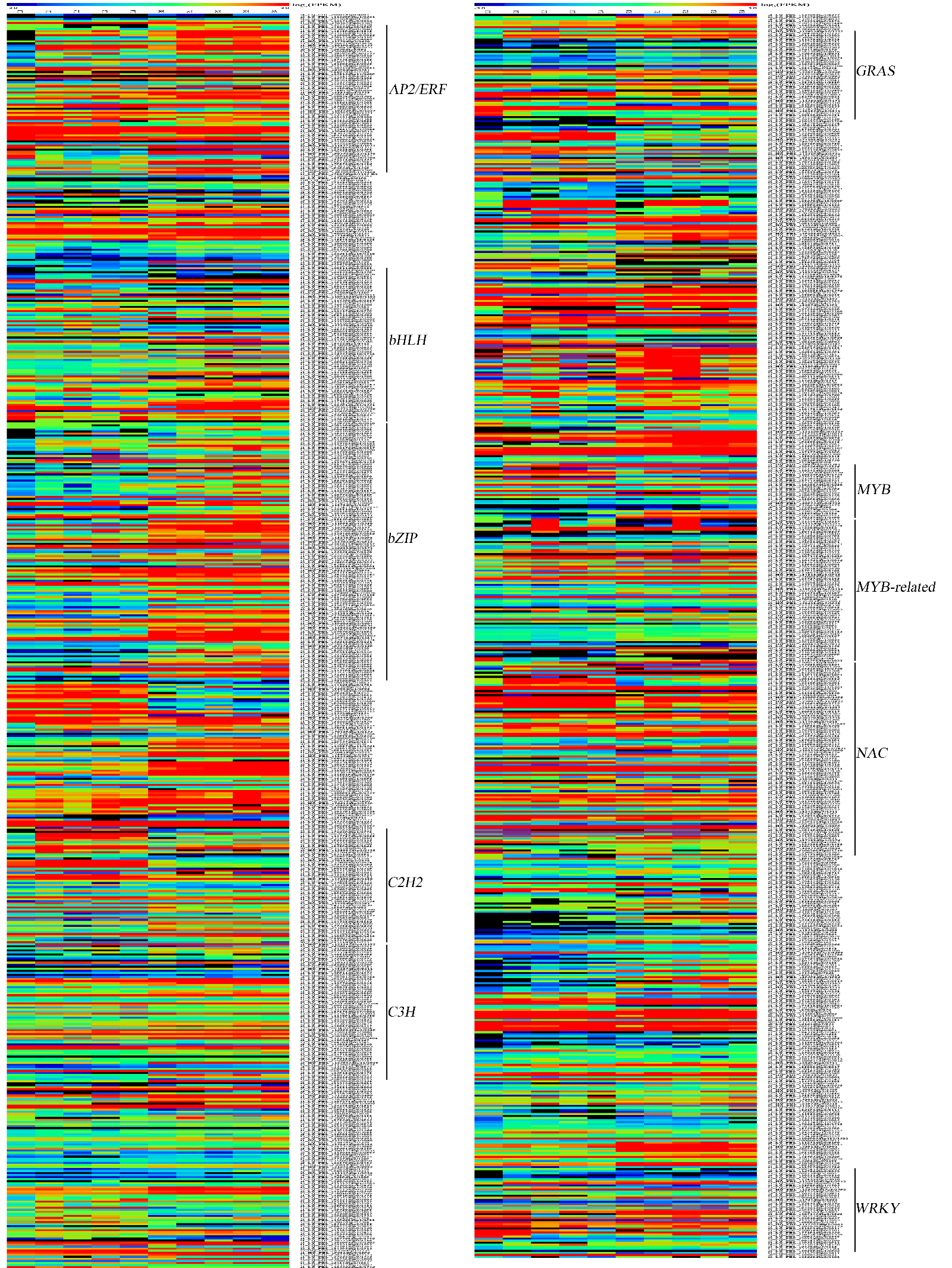


**Figure S7.** **Heatmap plot of the expression levels of the transcription factors.**

Note: The gene expression is based on the z-scores of log_2_(FPKM) value. The red and blue colors indicate high and low expression levels, respectively.


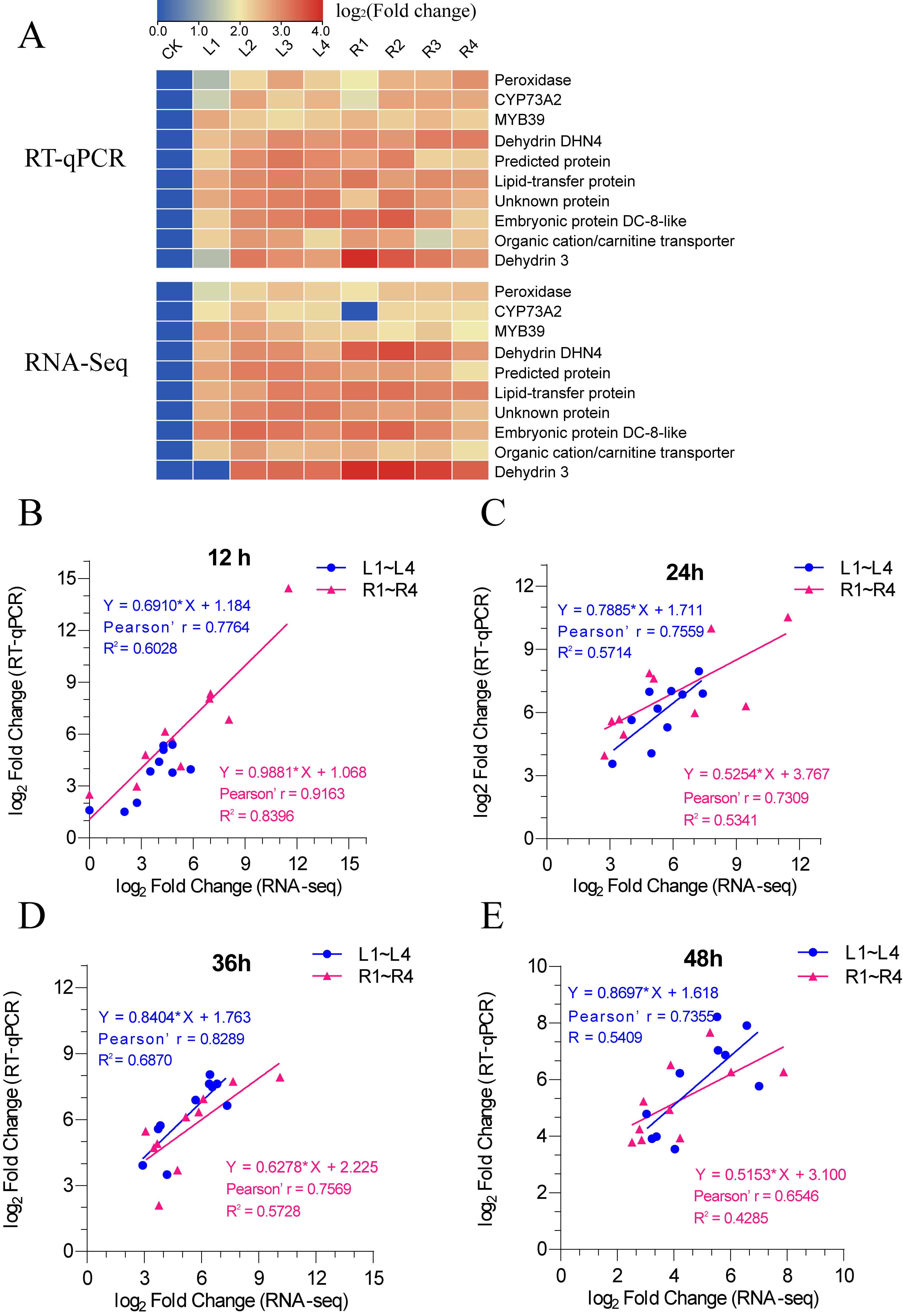


**Figure S8.** The expression pattern of ten selected DEGs after salt treatment was evaluated by RNA-seq and verified by RT-qPCR. **(A)** The heat map of log_2_(Fold Change) of RNA-seq and RT-qPCR. Figure **(B-E)** shows the correlation between RNA-seq and RT-qPCR of leaves and roots under salt stress.
